# Stereochemical Characterization of Bis-Indolyl Alkaloids from the Marine Fungus *Aspergillus ochraceopetaliformis* FM451 and Their Differential Effects on *Candida albicans*

**DOI:** 10.3390/md24100336

**Published:** 2026-09-27

**Authors:** Chen Huo, Hyejin Jeong, Hui Lei, KH Ahammad Uz Zaman, Yousong Ding, Shugeng Cao, Kyung-Tae Lee, Ki Hyun Kim

**Affiliations:** 1 School of Pharmacy, Sungkyunkwan University, Suwon 16419, Republic of Korea; huochen_0213@163.com; 2 Korea Zoonosis Research Institute, Jeonbuk National University, Iksan 54531, Republic of Korea; hj2022@jbnu.ac.kr; 3 Department of Pharmaceutical Sciences, Daniel K. Inouye College of Pharmacy, University of Hawai’i at Hilo, 200 W. Kawili St., Hilo, HI 96720, USA; huilei@hawaii.edu (H.L.); kzaman@hawaii.edu (K.A.U.Z.); 4 Department of Medicinal Chemistry, Center for Natural Products, Drug Discovery and Development, College of Pharmacy, University of Florida, Gainesville, FL 32610, USA; yding@cop.ufl.edu

**Keywords:** marine-derived fungi, *Aspergillus ochraceopetaliformis*, bis-indolyl alkaloids, chiral HPLC, antifungal activity, *Candida albicans*

## Abstract

Marine-derived fungi are a rich source of structurally diverse secondary metabolites with unique biological activities. Guided by an integrated GNPS molecular networking strategy, chemical investigation of the culture extract of the marine-derived fungus *Aspergillus ochraceopetaliformis* FM451 led to the isolation of four bis-indolyl alkaloids (**1**–**4**). Their structures were elucidated as asterriquinol G (**1**), asterriquinone K (**2**), asterriquinone C-1 (**3**), and asterriquinol E (**4**) based on comprehensive NMR spectroscopic analyses and HRESIMS data. The enantiomers of asterriquinol G (**1a** and **1b**) were further separated by semi-preparative chiral HPLC, and their absolute configurations were assigned by comparison of the experimental and calculated specific rotations. In addition, the absolute configuration of asterriquinone K (**2**) was assigned as 11″*R* based on its specific rotation. Although the planar structures of compounds **1** and **2** had previously been reported, 11″*R*-asterriquinol G (**1a**), 11″*S*-asterriquinol G (**1b**), and 11″*R*-asterriquinone K (**2**) represent the first complete stereochemical characterization of these bis-indolyl alkaloids. All isolated compounds were evaluated for their effects on the growth and biofilm-associated phenotypes of *Candida albicans*. Although their effects on fungal growth were limited, compound **1a** produced the greatest reduction in surface-associated biofilm biomass among the tested compounds, reducing crystal violet-stained biomass to approximately 50% of the control level at 16 μg/mL. Its enantiomer **1b** was associated with more pronounced alterations in residual biofilm morphology, with fewer apparent hyphal structures than those observed following treatment with **1a** at comparable concentrations. These findings provide preliminary evidence that the stereochemistry of asterriquinol G may differentially influence its biofilm-associated effects on *C. albicans* and provide a basis for further stereochemistry-oriented studies of bis-indolyl alkaloids from *A. ochraceopetaliformis* FM451.

## 1. Introduction

Fungi have long been recognized as prolific producers of structurally diverse secondary metabolites with remarkable pharmacological properties [1]. Numerous fungal metabolites have been successfully developed into pharmaceuticals and agrochemicals, underscoring the importance of fungi as an invaluable resource for drug discovery [2,3,4]. Among fungal genera, *Aspergillus*, comprising more than 340 recognized species, is particularly renowned for its ability to biosynthesize a wide variety of specialized metabolites, including alkaloids, terpenoids, xanthones, polyketides, and peptides [5,6,7]. These metabolites exhibit a broad spectrum of biological activities, including antimicrobial, antiviral, cytotoxic, insecticidal, neuroprotective, and antioxidant effects [8,9,10,11,12], making *Aspergillus* one of the most important sources of structurally diverse bioactive natural products.

Among the species within this genus, *Aspergillus ochraceopetaliformis* remains chemically underexplored. Although it has occasionally been isolated from clinical cases of onychomycosis in immunocompetent individuals, current evidence remains insufficient to establish it as a human pathogen [13,14,15]. Recent studies have revealed its potential to produce structurally unique and biologically active secondary metabolites. Guo et al. reported dizinchydroxyneoaspergillin, a dimeric zinc complex with potent antibacterial activity with MIC values ranging from 0.45 to 7.8 μg/mL [16], while Cong et al. described two pairs of sulfur-containing racemic natural products with significant anti-inflammatory activity [17]. More recently, cage-like 5/6/5-fused spirocyclic amides from *A. ochraceopetaliformis* SCSIO 05702 were found to inhibit viral entry and adsorption, demonstrating promising antiviral activity [18]. These findings suggest the considerable biosynthetic potential of *A. ochraceopetaliformis* and highlight this species as a promising source of structurally unique bioactive natural products.

The global rise of invasive fungal infections, together with the emergence of multidrug-resistant pathogens such as *Candida auris* and azole-resistant *Aspergillus fumigatus*, has become a major challenge in modern medicine [19]. Despite the growing clinical need, the current antifungal arsenal remains limited by a narrow spectrum of activity, host toxicity, and unfavorable pharmacokinetic properties [20]. These limitations continue to motivate the exploration of natural products as sources of diverse bioactive scaffolds and potential chemical probes for investigating fungal growth and virulence-related phenotypes [1,21]. Fungi are particularly prolific sources of such metabolites and have yielded numerous clinically important antimicrobial agents [22,23]. Accordingly, continued investigation of chemically underexplored fungal species may expand the chemical and stereochemical diversity of bioactive natural products and provide opportunities to investigate structure-dependent effects on fungal phenotypes. In this context, bis-indolyl alkaloids are of particular interest because their structural and stereochemical diversity may give rise to differences in their biological effects.

As part of our ongoing efforts to discover structurally novel bioactive natural products [24,25,26], we investigated the secondary metabolites of the marine-derived fungus *A. ochraceopetaliformis* FM451. GNPS molecular networking-guided chemical investigation of the fungal culture extract led to the isolation of four bis-indolyl alkaloids (**1**–**4**). Their structures were elucidated through comprehensive NMR spectroscopic analyses and HR-ESIMS data. A pair of enantiomers, **1a** and **1b**, was separated by chiral HPLC, and their absolute configurations were established by comparison of the experimental and calculated specific rotations. The absolute configuration of compound **2** was likewise assigned based on its specific rotation. Although the planar structures of compounds **1** and **2** had previously been reported, compounds **1a**, **1b**, and **2** represent the first complete stereochemical characterization of these bis-indolyl alkaloids. All isolated compounds were subsequently evaluated for their effects on *C. albicans* growth and biofilm-associated phenotypes. Herein, we report the isolation and structural characterization of these bis-indolyl alkaloids from *A. ochraceopetaliformis* FM451, together with an evaluation of their effects on *C. albicans*.

## 2. Results

### 2.1. GNPS Molecular Networking-Guided Isolation of Compounds (***1***–***4***)

To explore the secondary metabolite profile of the *A. ochraceopetaliformis* FM451 extract and prioritize metabolites for targeted isolation, the extract was first subjected to LC–MS/MS analysis. The acquired MS/MS data were processed using MZmine and subsequently analyzed by molecular networking through the GNPS2 platform. The resulting molecular network enabled visualization of MS/MS spectral similarities among the detected features and revealed several molecular families comprising structurally related metabolites.

Among these molecular families, a cluster containing precursor ions at *m*/*z* 467.198 [M + H]^+^ and *m*/*z* 483.228 [M + H]^+^ attracted particular attention. The feature at *m*/*z* 467.198 [M + H]^+^ was subjected to SIRIUS-based in silico analysis, and comparison of its experimental MS/MS spectrum with predicted fragmentation patterns supported its putative annotation as asterriquinone C-1 (Figure 1). Notably, the feature at *m*/*z* 483.228 [M + H]^+^ exhibited a similar fragmentation pattern and clustered closely with the *m*/*z* 467.198 [M + H]^+^ feature, suggesting a close structural relationship between the two metabolites. This molecular family was therefore prioritized for targeted isolation. Subsequent LC–MS-guided fractionation and chromatographic purification of the fermentation extract led to the isolation of four bis-indolyl alkaloids, designated as compounds **1**–**4** (Figure 2). Interestingly, compounds **1** and **2**, although structurally related to the metabolites in the targeted molecular family, appeared as individual singleton nodes rather than as members of this cluster in the GNPS molecular network (Figure 1).

### 2.2. Structural Elucidation of Compounds

Compound **1** was isolated as a yellow powder. Its molecular formula was established as C_36_H_42_N_2_O_6_ based on HR-ESIMS data. The ^1^H NMR spectrum displayed resonances attributable to two indole moieties with characteristic aromatic proton patterns, a terminal vinyl group, four methoxy groups, and two gem-dimethyl groups. The aromatic proton resonances were consistent with the substitution patterns of the two indole moieties reported for asterriquinol G, previously isolated from *Aspergillus* sp. SCSIO 41018 [27]. In addition, the characteristic proton signals of the terminal vinyl group supported the presence of a 2-methylbut-3-en-2-yl substituent on one indole moiety, whereas the remaining aliphatic and oxygenated proton resonances were consistent with a 3-hydroxy-2-methoxy-3-methylbutyl substituent on the other indole moiety. Comparison of the NMR and HR-ESIMS data with the reported values identified compound **1** as asterriquinol G (Figures S2–S4). However, compound **1** exhibited a specific rotation of zero (${\left[\alpha \right]}_{D}^{20}$ = 0), indicating that it was isolated as a racemic mixture. Notably, the absolute configuration at C-11″ has not been determined for asterriquinol G in the previous report.

To resolve the enantiomers and determine their absolute configurations, compound **1** was separated by semi-preparative chiral HPLC on a Chiralpak IE3 column using *n*-hexane–EtOH (93:7, *v*/*v*) as the mobile phase, affording pure compounds **1a** and **1b** (Figure 3). Compound **1a** exhibited a positive optical rotation, ${\left[\alpha \right]}_{D}^{20}$ +58.0 (*c* 0.01, MeOH), whereas compound **1b** showed a negative optical rotation, ${\left[\alpha \right]}_{D}^{20}$ −23.5 (*c* 0.03, MeOH). Their opposite optical rotations confirmed that **1a** and **1b** constitute an enantiomeric pair. As enantiomers, compounds **1a** and **1b** are expected to exhibit identical ^1^H NMR spectra to that of racemic compound **1** under achiral experimental conditions. Therefore, their absolute configurations were investigated by comparing experimental and theoretically calculated specific rotations following chiral HPLC separation. The positive optical rotation of **1a** was consistent with that reported for asterriquinone I (${\left[\alpha \right]}_{D}^{20}$ +20.0, *c* 0.04, MeOH), suggesting the same 11″*R* absolute configuration [27]. To further support this assignment, specific rotations were calculated for all optimized conformers at the B3LYP/6-31G(d,p) level and Boltzmann averaged according to their relative populations. The calculated specific rotations (${\left[\alpha \right]}_{D}^{20}$ +73.0 for **1a** and −14.9 for **1b**) were in good agreement with the experimental values, supporting the assignment of **1a** and **1b** as 11″*R*-asterriquinol G and 11″*S*-asterriquinol G, respectively (Table S1).

The other compounds were identified as asterriquinone K (**2**) [27], asterriquinone C-1 (**3**) [27], and asterriquinol E (**4**) [28] by comparison of their NMR spectroscopic and HRESIMS data with those reported in the literature (Figures S7–S12). The absolute configuration of compound **2** at C-11″ was assigned as 11″*R* based on its positive specific rotation, ${\left[\alpha \right]}_{D}^{20}$ +24.8 (*c* 0.03, MeOH), which was consistent with the reported value for asterriquinone I [27].

### 2.3. Effects of the Compounds on Candida albicans Growth and Biofilm-Associated Phenotypes

Given the strong biofilm-forming capacity and morphogenetic plasticity of *Candida albicans*, the effects of the isolated compounds on fungal growth and biofilm-associated phenotypes were evaluated under biofilm-inducing conditions. *C. albicans* cells were cultured in RPMI medium under static conditions in the presence of serially diluted compounds. Following 48h of incubation, relative fungal growth and crystal violet-stained surface-associated biomass were quantified. Among the tested compounds (**1a**, **1b**, and **2**–**4**), compound **1a** produced the greatest, although modest, reduction in fungal growth, with the most pronounced effect observed at 128 μg/mL (Figure 4A). Consistent with this observation, compound **1a** also produced the greatest reduction in crystal violet-stained attached biomass compared with the other compounds (Figure 4B). However, none of the compounds demonstrated a strictly dose-dependent decrease in biomass. Instead, partial recovery of crystal violet signals was observed at higher concentrations for all compounds, resulting in a non-monotonic response pattern.

To further examine these phenotypes, residual attached structures remaining after washing were visualized by crystal violet staining and light microscopy (Figure 4C). Overall, most compounds appeared to suppress hyphal transition at the highest tested concentration (128 μg/mL). However, hyphal structures began to reappear in most treatment groups at 64 μg/mL. Interestingly, compound **1b** showed a distinct morphological phenotype, in which hyphal transition was not clearly observed even at 32 μg/mL, while hyphal structures began to emerge at 16 μg/mL. In contrast, compound **1a** treatment was associated with a greater reduction in total attached biomass rather than strong suppression of morphological transition.

These findings suggest that inhibition of hyphal differentiation and reduction in total attached biomass are not necessarily correlated in response to treatment of compounds. Because crystal violet staining reflects total residual surface-associated biomass rather than hypha-specific structures, the apparent recovery of biomass signals at higher concentrations may partially result from the retention or accumulation of attached yeast-form cells following suppression of filamentation. Collectively, these results indicate that the isolated compounds differentially modulate fungal growth, surface attachment, and morphogenetic transition, suggesting potentially distinct antibiofilm-associated modes of action among the compounds.

## 3. Discussion

Enantiomeric natural products are widely distributed in nature and frequently exhibit distinct biological activities owing to their differential recognition by chiral biological targets [29,30]. Previous studies have demonstrated that even subtle stereochemical differences can markedly influence biological activities despite identical planar structures [31,32,33]. For example, the furoquinoline alkaloid enantiomers (±)-zanthonitidine A exhibited different antibacterial activities against *Enterococcus faecalis*, with the (−)-enantiomer being approximately twofold more active than the (+)-enantiomer [33]. Likewise, only the (+)-enantiomer of the δ-lactone 6-styryl-7,8-dihydroxy-4-methoxypyran-2-one exhibited antifungal activity against *Candida albicans*, whereas its antipodal enantiomer was inactive [34]. These examples highlight the critical role of stereochemistry in determining the biological properties of natural products. In this context, the chiral separation and absolute configurational assignment of asterriquinol G reported herein provide a structural basis for investigating the stereochemistry-dependent biological activities of this class of fungal metabolites.

Consistent with this premise, the enantiomeric pair **1a** and **1b** exhibited partially distinct phenotypic profiles against *Candida albicans* despite sharing an identical planar structure. Compound **1a** showed stronger inhibition of fungal growth and crystal violet-stained surface-associated biomass, whereas compound **1b** tended to exert a greater inhibitory effect on hyphal morphogenesis. Rather than differing solely in the magnitude of their effects, the two enantiomers appeared to preferentially influence distinct aspects of *C. albicans* biology, suggesting that stereochemistry may differentially modulate fungal growth, surface-associated biomass, and biofilm-associated morphology. These findings further indicate that stereochemical variation can influence the phenotypic effects of fungal natural products, even when their planar structures are identical. One plausible explanation for these differences is stereoselective recognition by fungal cellular targets, as enantiomers can interact differently with chiral biological components [33,34]. In *C. albicans*, such stereoselective interactions may differentially influence cellular processes involved in adhesion and morphogenetic transition, potentially contributing to the distinct effects of **1a** and **1b** on surface-associated biomass and hyphal morphology. However, the present phenotypic data do not permit the identification of a specific molecular target or signaling pathway. Further target-based and mechanistic studies are therefore required to elucidate the molecular basis of these stereochemistry-dependent effects.

None of the tested compounds produced a strictly dose-dependent reduction in crystal violet-stained biofilm biomass. This observation should be interpreted with caution because crystal violet staining reflects the total surface-associated biomass rather than fungal viability [35]. Moreover, *C. albicans* biofilms are heterogeneous structures comprising yeast-form cells, hyphal elements, and extracellular material, which can vary independently in response to treatment [36,37,38]. Accordingly, the observed non-monotonic response may reflect changes in cellular attachment, morphology, extracellular material, or overall biofilm architecture rather than a proportional change in fungal growth. Nevertheless, the different patterns observed for **1a** and **1b** suggest that the absolute configuration of asterriquinol G may influence its phenotypic effects on fungal growth, attached biomass, and residual biofilm morphology. Although the molecular basis of these differences remains unclear, the present findings extend the stereochemical characterization of asterriquinol G to its biological evaluation.

The possibility that asterriquinone K (**2**) is an isolation artifact was also considered. Several structurally related asterriquinones have previously been reported as fungal secondary metabolites [39,40,41], providing context for its possible natural origin. To investigate whether **2** might have formed during chromatographic isolation, the crude fungal extract stored under refrigerated conditions was reanalyzed by LC–HRMS. The LC–HRMS reanalysis revealed a peak corresponding to **2** at a retention time of 12.301 min, with an accurate mass of *m*/*z* 583.2812. Both the retention time and accurate mass were consistent with those of isolated compound **2** analyzed under identical conditions (Figure S13). These findings support the presence of **2** in the crude extract before chromatographic purification and argue against its formation solely during the subsequent isolation procedures.

Beyond clarifying the origin of compound **2**, further investigation is needed to establish the biological relevance of the observed activities. In particular, the cytotoxicity of these compounds toward mammalian cells was not evaluated in the present study, and their selectivity therefore remains to be established. Further studies incorporating quantitative yeast-to-hypha transition analysis, metabolic viability assays, mammalian-cell cytotoxicity and selectivity assessments, and target-based approaches will be required to determine whether **1a** and **1b** differ in potency, cellular exposure, target engagement, or effects on morphogenetic signaling, and whether their biofilm-associated effects can be achieved at concentrations that are not cytotoxic to mammalian cells. Overall, these findings provide a basis for further stereochemistry-oriented structure–activity studies of bis-indolyl alkaloids.

## 4. Materials and Methods

### 4.1. General Experimental Procedures

Optical rotations were measured using a Jasco P-2000 polarimeter (Jasco, Easton, MD, USA). Nuclear magnetic resonance (NMR) spectra were recorded on a Bruker AVANCE III HD spectrometer operating at 850 MHz (Bruker, Karlsruhe, Germany) at the National Center for Inter-University Research Facilities (NCIRF), Seoul National University (Seoul, Republic of Korea). Chemical shifts (δ) are reported in ppm, and coupling constants (*J*) are given in Hz. Semi-preparative HPLC was performed using a Waters 1525 binary pump system coupled with a Waters 996 photodiode array detector (Waters Corporation, Milford, MA, USA) and equipped with a Phenomenex Luna C18 column (250 × 10 mm, 10 μm; flow rate, 2.0 mL/min; Phenomenex, Torrance, CA, USA). Chiral separations were performed on a Chiralpak IE3 column (4.6 × 250 mm, 3 μm; flow rate, 1.0 mL/min; Daicel Chiral Technologies, Osaka, Japan). LC–MS analyses were performed using an Agilent 1200 Series HPLC system equipped with a diode-array detector and a 6130 Series ESI mass spectrometer (Agilent Technologies, Santa Clara, CA, USA), fitted with a Kinetex C18 100 Å analytical column (100 × 2.1 mm, 5 μm; flow rate, 0.3 mL/min; Phenomenex, Torrance, CA, USA). High-resolution ESI-MS and ESI-MS/MS data were acquired using an Agilent 6545 quadrupole time-of-flight (Q-TOF) mass spectrometer coupled to an Agilent 1290 Infinity II HPLC system (Agilent Technologies, Santa Clara, CA, USA). Reversed-phase C18 silica gel (230–400 mesh; Merck, Darmstadt, Germany) and Diaion HP-20 resin (Alfa Aesar, Ward Hill, MA, USA) were used for column chromatography. Thin-layer chromatography (TLC) was carried out on precoated silica gel 60 F254 and RP-18 F254S plates (Merck, Darmstadt, Germany). TLC spots were visualized under UV light (254 and 365 nm) or by heating after spraying with anisaldehyde–sulfuric acid reagent.

### 4.2. Reagents and Chemicals

Analytical-grade solvents used for extraction and solvent partitioning were purchased from Samchun Chemical Co., Ltd. (Seoul, Republic of Korea). HPLC-grade solvents used for chromatographic separation and HPLC purification were purchased from Honeywell International Inc. (Charlotte, NC, USA). Unless otherwise specified, all solvents, chemicals, and reagents were commercially available and used as received without further purification.

### 4.3. Fungal Material

The fungal strain FM451, identified as *A. ochraceopetaliformis*, was isolated from a marine sediment sample collected at Hanauma Bay, Oahu, Hawaii, USA, in January 2015 (Figure S1). The strain was identified based on sequencing of the internal transcribed spacer (ITS) region of the rDNA, followed by comparison with sequences available in the GenBank database using the NCBI BLAST web service. The strain has been preserved at −80 °C at the Daniel K. Inouye College of Pharmacy, University of Hawaii at Hilo (Hilo, HI, USA). The rDNA ITS1–ITS4 sequence of this strain has been deposited in GenBank under accession no. PP395677.

### 4.4. Fermentation

The fungal strain FM451 was reactivated from a −80 °C stock culture on potato dextrose agar (PDA) plates and incubated at 28 °C for 3 days. Agar plugs containing actively growing mycelia were then inoculated into potato dextrose broth (PDB) supplemented with marine salt (Instant Ocean^®^, Blacksburg, VA, USA; 15 g/L). Fermentation was carried out under static conditions at room temperature (24 °C) under ambient light for 4 weeks.

### 4.5. LC-MS/MS Analysis and Molecular Networking

The crude extract of *A. ochraceopetaliformis* FM451 was analyzed by HPLC-ESI-HRMS/MS using an Agilent 6545B QTOF mass spectrometer (Agilent Technologies, Santa Clara, CA, USA) equipped with an ACQUITY UPLC BEH C18 column (2.1 × 150 mm, 1.7 μm; Waters Corporation, Milford, MA, USA) at Sungkyunkwan University. Sample was prepared at 2 mg/mL in HPLC-grade solvent, and 3 μL was injected. The column was maintained at 30 °C and operated at a flow rate of 0.3 mL/min. The mobile phases consisted of 0.1% formic acid in water (A) and acetonitrile (B), with a gradient of 10–100% B over 15 min, followed by 2 min at 100% B and re-equilibration to 10% B for 3 min. Mass spectra were acquired in positive ion mode over an *m*/*z* range of 50–1700. The source parameters were set as follows: gas temperature, 320 °C; drying gas flow, 8 L/min; sheath gas temperature, 350 °C; sheath gas flow, 11 L/min; nebulizer pressure, 35 psi; capillary voltage, 3500 V; skimmer voltage, 65 V; octopole RF voltage, 750 V; and fragmentor voltage, 150 V. MS/MS spectra were acquired at 3 spectra/s using collision energies of 20 and 40 eV, with up to five precursor ions selected per cycle and active exclusion set to 0.2 min. Data acquisition was performed using Agilent MassHunter Workstation Software (version B.09.00; Agilent Technologies).

Raw LC–MS/MS data files (.raw) were processed using MZmine 4.6.1 [42]. The resulting “quant” and “.mgf” files were uploaded to the GNPS2 platform (https://gnps2.org/) for analysis via the feature-based molecular networking FBMN workflow [43]. The precursor ion and fragment ion mass tolerances were both set to 0.02 Da, with a minimum cosine similarity score of 0.5 and a minimum of three matched fragment peaks. All other parameters were maintained at their default settings. The resulting molecular network was visualized using Cytoscape 3.9.1 [44]. For further structural annotation of selected molecular features, the corresponding MS/MS spectra were analyzed using SIRIUS 6.3.7 [45].

### 4.6. Extraction and Isolation

After cultivation, the cultures were filtered through cotton to separate the culture broth from the fungal mycelia. The mycelia were extracted three times with acetone, and the combined extracts were concentrated under reduced pressure to remove the organic solvent, yielding a crude extract. The extract was combined with the culture broth and subjected to Diaion HP-20 column chromatography, followed by stepwise elution with a MeOH–H_2_O gradient (50%, 90%, and 100% MeOH) to afford three fractions, designated FM0 (3.5 g), FM1 (5.6 g), and FM2 (0.5 g). A portion of fraction FM1 (3.0 g of the total 5.6 g), eluted with 90% MeOH–H_2_O, was subjected to reversed-phase C18 silica gel column chromatography using a stepwise MeOH–H_2_O gradient to afford 10 fractions (FM11–FM110) based on TLC analysis. Fraction FM18 (40.0 mg) was purified by semi-preparative RP-HPLC (30–42% MeCN/H_2_O, 2.0 mL/min) to yield compound **1** (1.5 mg, *t*_R_ = 83.0 min), together with compounds **2** (0.6 mg, *t*_R_ = 70.5 min), **3** (1.1 mg, *t*_R_ = 60.0 min), and **4** (0.7 mg, *t*_R_ = 74.0 min). The compound **1** (1.5 mg) was further separated by semi-preparative chiral HPLC on a Chiralpak IE3 column using *n*-hexane–EtOH (93:7, *v*/*v*) as the mobile phase to afford compound **1a** (0.2 mg, *t*_R_ = 50.5 min) and compound **1b** (0.6 mg, *t*_R_ = 53.5 min).

#### 4.6.1. 11″*R*-Asterriquinol G (**1a**)

Yellow powder: ${\left[\alpha \right]}_{D}^{20}$ +58.0 (*c* 0.01, MeOH); UV (MeOH) *λ*_max_ (log *ε*) 204 (2.7), 224 (2.8), 285 (2.3) nm. HR–ESIMS (positive–ion mode) *m*/*z* 599.3116 [M + H]^+^ (calculated for C_36_H_43_N_2_O_6_, 599.3116).

#### 4.6.2. 11″*S*-Asterriquinol G (**1b**)

Yellow powder: ${\left[\alpha \right]}_{D}^{20}$ −23.5 (*c* 0.03, MeOH); UV (MeOH) *λ*_max_ (log *ε*) 204 (2.7), 224 (2.8), 285 (2.3) nm. HR–ESIMS (positive–ion mode) *m*/*z* 599.3117 [M + H]^+^ (calculated for C_36_H_43_N_2_O_6_, 599.3116).

#### 4.6.3. 11″*R*-Asterriquinone K (**2**)

Dark purple powder: ${\left[\alpha \right]}_{D}^{20}$ +24.8 (*c* 0.03, MeOH); UV (MeOH) *λ*_max_ (log *ε*) 204 (2.4), 224 (2.2), 285 (2.6) nm. HR–ESIMS (positive–ion mode) *m*/*z* 583.2814 [M + H]^+^ (calculated for C_36_H_43_N_2_O_6_, 583.2803).

### 4.7. Specific Rotation Calculations

To support the assignment of the absolute configurations of compounds **1a** and **1b**, an initial conformational search was performed using the OPLS4 force field implemented in MacroModel (v2024-4, Schrödinger LLC, New York, NY, USA) with a mixed torsional/low-mode sampling protocol. The conformational search was carried out in the gas phase using an energy window of 20 kJ/mol. All generated conformers were subsequently optimized at the B3LYP/6-31G(d,p) level using TmoleX (version 4.3.2, Turbomole GmbH, Karlsruhe, Germany). Specific rotations were calculated at the B3LYP/6-31G(d,p) level at the sodium D-line (589 nm), and the final values were obtained by Boltzmann averaging of all optimized conformers [46,47].

### 4.8. Antifungal Susceptibility and Biofilm Formation Assays

Antifungal susceptibility and biofilm formation assays were performed using a modified broth microdilution method based on the Clinical and Laboratory Standards Institute (CLSI) guidelines [48]. Candida albicans SC5314 fungal cells were cultured overnight on YPD agar plates, collected, washed twice with sterile phosphate-buffered saline (PBS), and adjusted to an optical density at 600 nm (OD600) of 0.01 in RPMI 1640 medium buffered with MOPS. The isolated compounds were prepared as stock solutions in DMSO. For twofold serial dilution, 5 μL of each compound stock solution was added to 195 μL of the fungal suspension in the first well of a flat-bottomed 96-well microplate. After thorough mixing, 100 μL was transferred sequentially into adjacent wells containing 100 μL of the fungal suspension. Following mixing of the final well, 100 μL was discarded, resulting in a final volume of 100 μL per well and compound concentrations ranging from 1 to 128 μg/mL. Vehicle controls were prepared using the same serial dilution procedure without compound. Plates were incubated at 35 °C for 48 h under static conditions. Following incubation, fungal growth was quantified by measuring OD600 using a microplate reader. Relative growth (%) was calculated by normalizing treated samples to the untreated control group. After OD measurement, the culture supernatants containing planktonic and non-adherent cells were carefully removed. Wells were gently washed twice with sterile PBS to remove unattached cells, leaving only the surface-associated biofilm structures. The remaining attached biofilms were stained with 2% crystal violet solution for 30 min at room temperature. Excess stain was removed by washing with distilled water, and the plates were completely air-dried.

For qualitative assessment of biofilm morphology, crystal violet-stained residual biofilms were directly imaged under a light microscope after drying. Representative images were acquired at identical magnifications and exposure settings. For quantitative biofilm analysis, bound crystal violet was solubilized using 100% ethanol for 30 min, and absorbance was measured at 590 nm using a microplate reader. Relative biofilm formation (%) was calculated by normalizing each treated sample to the untreated control group.

### 4.9. Statistical Analysis

All experiments were independently performed at least three times. Data are presented as mean ± standard deviation (SD). Statistical significance was determined using one-way analysis of variance (ANOVA) followed by multiple comparison tests as indicated in the figure legend.

## 5. Conclusions

GNPS molecular networking-guided chemical investigation of the marine-derived fungus *A. ochraceopetaliformis* FM451 led to the isolation of four bis-indolyl alkaloids (**1**–**4**). The enantiomers of asterriquinol G were further separated by semi-preparative chiral HPLC, and their absolute configurations were assigned as 11″*R*-asterriquinol G (**1a**) and 11″*S*-asterriquinol G (**1b**). Together with the assignment of 11″*R*-asterriquinone K (**2**), this study provides the first complete stereochemical characterization of these bis-indolyl alkaloids. Compounds **1a**, **1b**, and **2**–**4** were evaluated for their effects on *Candida albicans* growth and biofilm-associated phenotypes. Among them, **1a** produced the greatest, although modest, reduction in fungal growth and crystal violet-stained attached biomass, whereas **1b** was associated with distinct alterations in residual biofilm morphology. Although further quantitative and mechanistic studies are required, the partially different phenotypic profiles of the two enantiomers suggest that the absolute configuration of asterriquinol G may influence its biological effects on *C. albicans*. Overall, this study demonstrates that marine-derived *A. ochraceopetaliformis* is a valuable source of structurally unique bis-indolyl alkaloids and highlights the potential role of stereochemistry in influencing their effects on fungal growth, attached biofilm biomass, and biofilm-associated morphology.

## Figures and Tables

**Figure 1:**
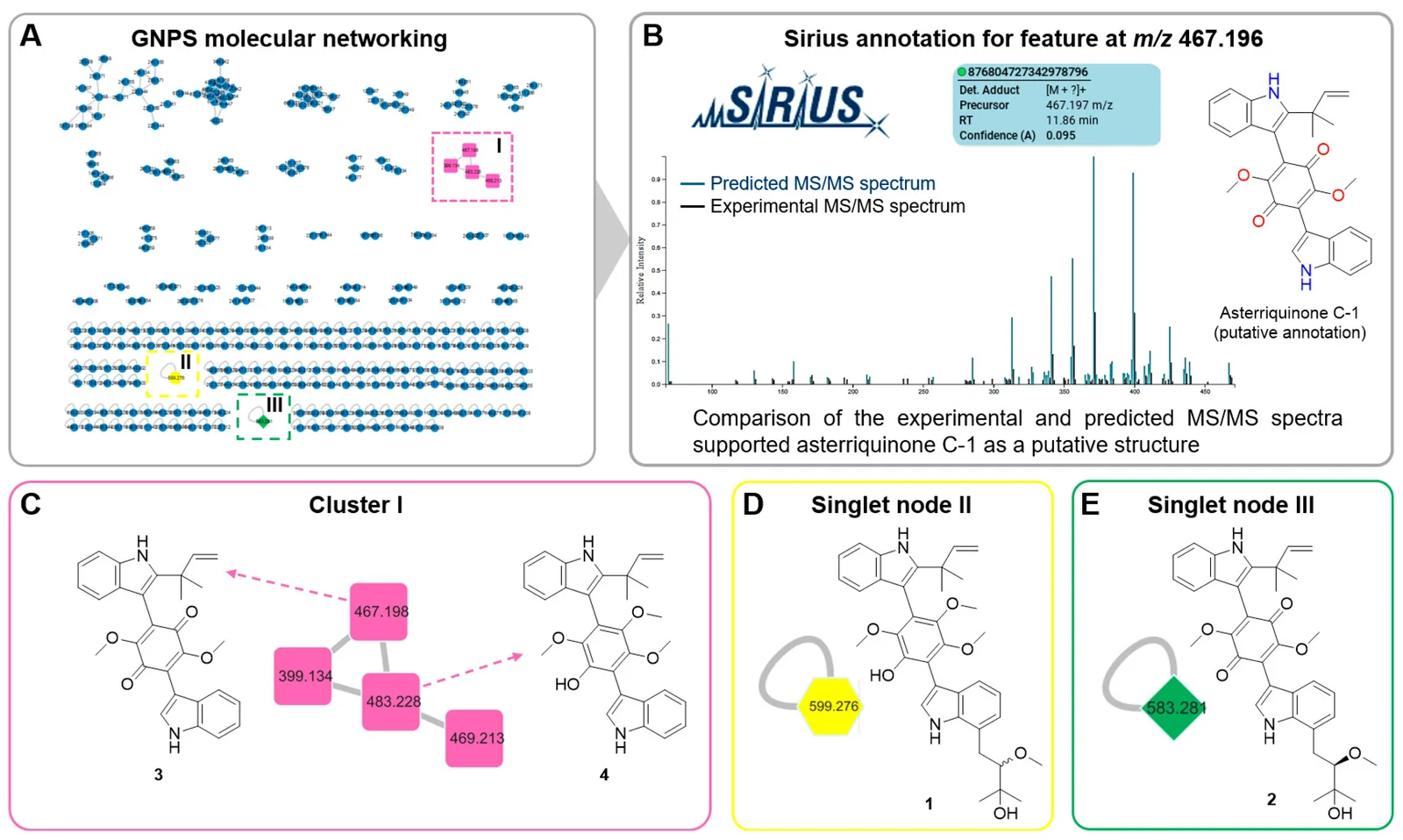
GNPS molecular networking-guided prioritization and targeted isolation of bis-indolyl alkaloids from *A. ochraceopetaliformis* FM451. (**A**) Full GNPS molecular network. (**B**) Comparison of the experimental MS/MS spectrum (black) of the molecular feature at *m*/*z* 467.198 with the SIRIUS-predicted MS/MS spectrum (blue), supporting its putative annotation as asterriquinone C-1. (**C**) Molecular family prioritized for targeted isolation based on MS/MS spectral similarity. (**D**,**E**) Singleton features at *m*/*z* 599.276 and 583.281, which were subsequently prioritized for targeted isolation and identified as compounds **1** and **2**, respectively.

**Figure 2:**
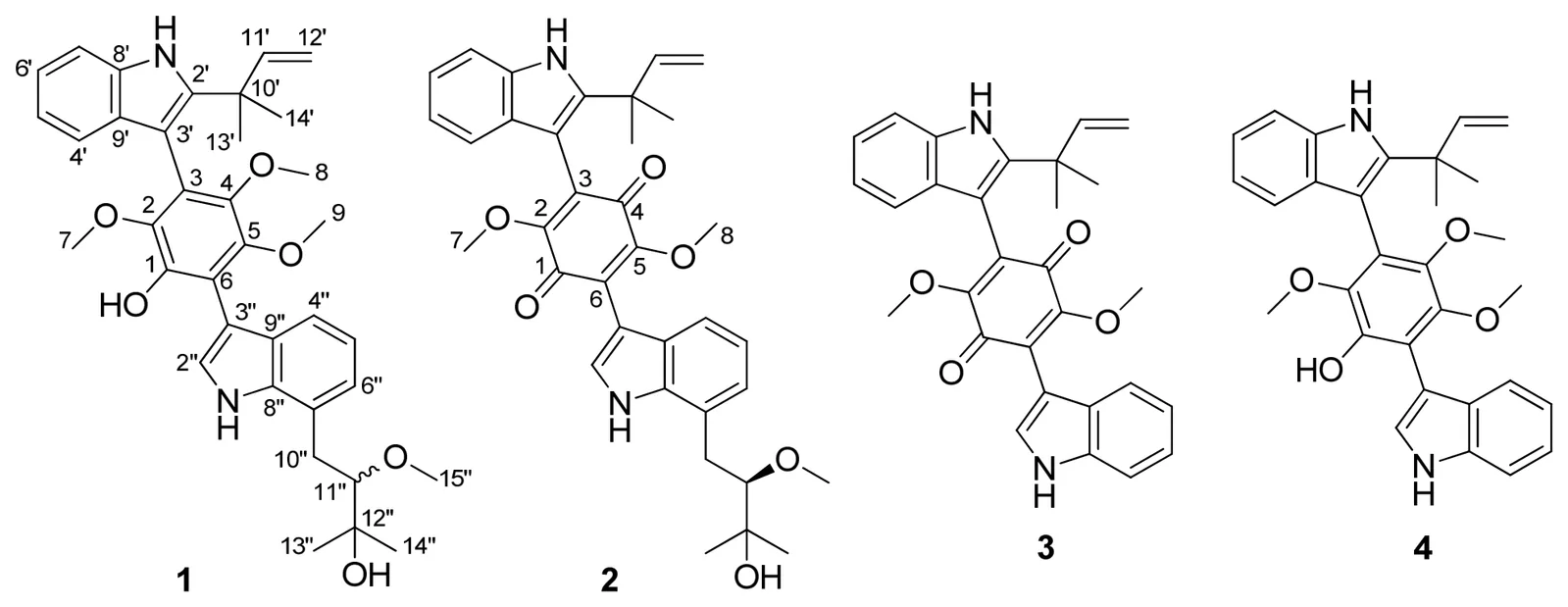
The chemical structures of compounds **1**–**4**.

**Figure 3:**
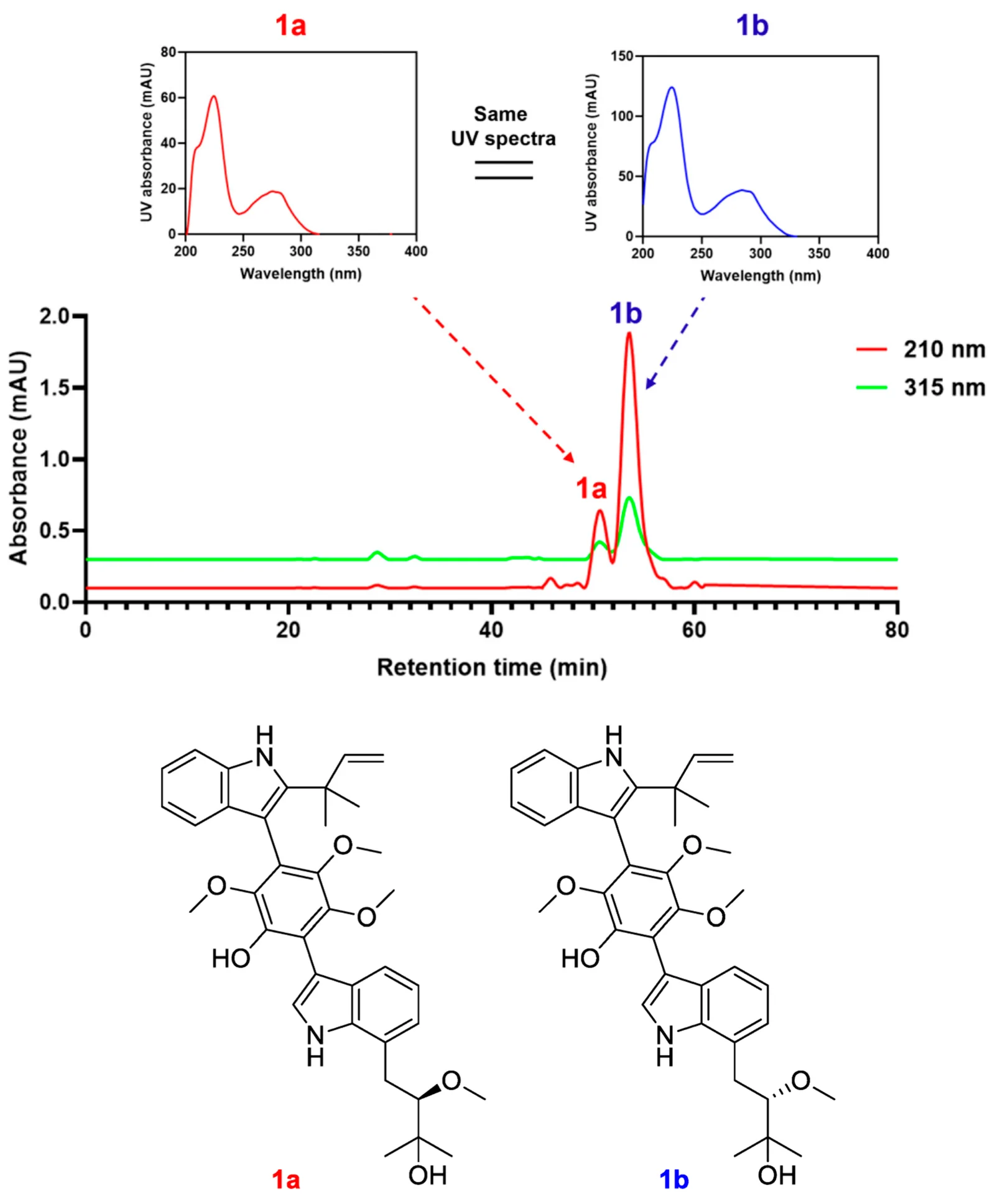
Chiral HPLC chromatogram showing the separation of compound **1** into enantiomers **1a** and **1b**.

**Figure 4:**
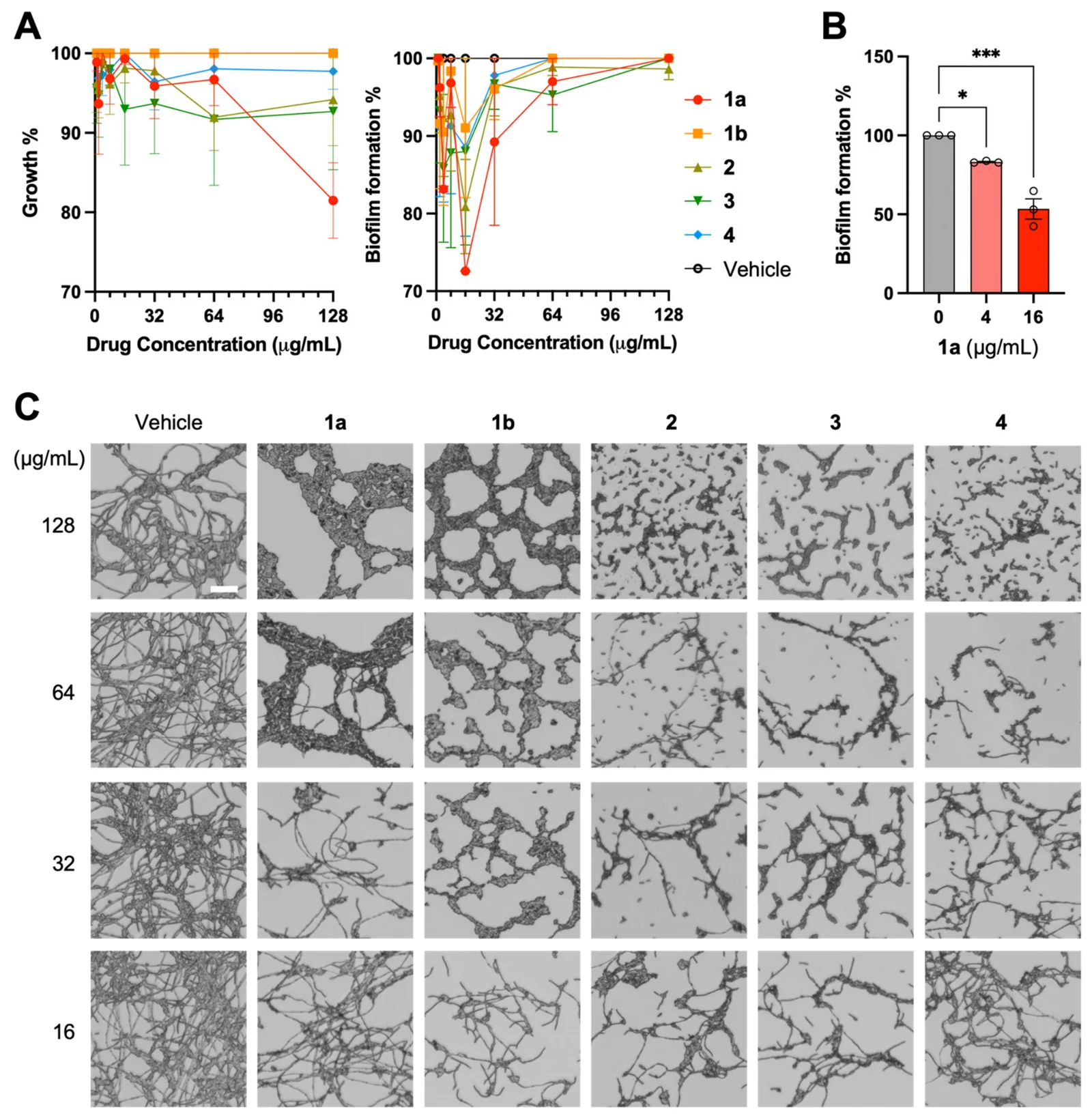
Differential effects of the isolated compounds on fungal growth, biofilm formation, and hyphal morphology. (**A**) Relative fungal growth and crystal violet-stained surface-associated biomass following treatment with compounds **1a**, **1b**, and **2**–**4** at the indicated concentrations. (**B**) Comparison of crystal violet-stained surface-associated biomass following treatment with compound **1a** at 4 and 16 μg/mL. Values in (**A**,**B**) were normalized to the vehicle-treated control (100%). Data presented as mean ± SEM from three independent experiments. Statistical significance was determined using one-way ANOVA followed by Dunnett’s multiple comparison test against the untreated control (* *p* < 0.05, *** *p* < 0.001). (**C**) Representative light microscopy images of residual surface-associated structures following compound treatment. Non-adherent cells were removed by washing, and the remaining structures were stained with crystal violet and air-dried prior to imaging. Scale bar = 50 μm.

## Data Availability

The data supporting this study’s findings are available from the corresponding author upon reasonable request.

## Supplementary Materials

The following supporting information can be downloaded at https://www.mdpi.com/article/10.3390/md24100336/s1: Table S1: Comparison of experimental and calculated specific rotations; Figure S1: Colony morphology of *Aspergillus ochraceopetaliformis* FM451 cultured on PDA medium; Figure S2: HR-ESIMS data of compound **1**; Figure S3: UV chromatogram of LC/MS for compound **1**; Figure S4: ^1^H NMR (CD_3_OD, 850 MHz) spectrum of compound **1**; Figure S5: HR-ESIMS data of compound **1a**; Figure S6: HR-ESIMS data of compound **1b**; Figure S7: HR-ESIMS data of compound **2**; Figure S8: ^1^H NMR (CD_3_OD, 850 MHz) spectrum of compound **2**; Figure S9: HR-ESIMS data of compound **3**; Figure S10: ^1^H NMR (CD_3_OD, 850 MHz) spectrum of compound **3**; Figure S11: HR-ESIMS data of compound **4**; Figure S12: ^1^H NMR (CD_3_OD, 850 MHz) spectrum of compound **4**; Figure S13: LC–HRMS analysis of compound **2** in the crude extract of *A. ochraceopetaliformis* FM451.
