# Supplementary material for: Stereochemical Characterization of Bis-Indolyl Alkaloids from the Marine Fungus Aspergillus ochraceopetaliformis FM451 and Their Differential Effects on Candida albicans
Source: Mar Drugs. 2026 Sep 27;24(10):336. doi: 10.3390/md24100336 (PMC13706091; doi:10.3390/md24100336)
Supplement: Supplementary file 1 [file marinedrugs-24-00336-s001.zip › marinedrugs-4550113-supplementary.pdf]

■ **Supplementary Material Contents:**

Table S1. Comparison of experimental and calculated specific rotations................................2

Figure S1. Colony morphology of *Aspergillus ochraceopetaliformis* FM451 cultured on PDA medium 2

Figure S2. HR-ESIMS data of compound **1**...2

Figure S3. UV chromatogram of LC/MS for compound **1**...3

Figure S4. ^{1}H-NMR (CD_3OD, 850 MHz) spectrum of compound **1** 3

Figure S5. HR-ESIMS data of compound **1a**...4

Figure S6. HR-ESIMS data of compound **1b** 4

Figure S7. HR-ESIMS data of compound **2**...5

Figure S8. ^{1}H NMR spectrum (CD_3OD, 850 MHz) of compound **2**...5

Figure S9. HR-ESIMS data of compound **3**...6

Figure S10. ^{1}H NMR spectrum (CD_3OD, 850 MHz) of compound **3**..6

Figure S11. HR-ESIMS data of compound **4**..7

Figure S12. ^{1}H NMR spectrum (CD_3OD, 850 MHz) of compound **4**..7

Figure S13. LC–HRMS analysis of compound **2** in the crude extract of *A. ochraceopetaliformis* FM451. (**A**) Total ion chromatogram (TIC) of the crude extract. (**B**) Extracted ion chromatogram (EIC) at *m/z* 583.2814 for compound **2**. (**C**) HRMS spectrum of peak at 12.301 min in the crude extract, and (**D**) HRMS spectrum of compound **2**. 8

Table S1. Comparison of experimental and calculated specific rotations.

Compound	Configuration	Experimental $[\alpha]_D^{20}$	Calculated $[\alpha]_D^{20}$
**1a**	11''*R*	$[\alpha]_D^{20}$ +58.0 (*c* 0.01, MeOH)	+73.0
**1b**	11''*S*	$[\alpha]_D^{20}$ -23.5 (*c* 0.03, MeOH)	-14.9

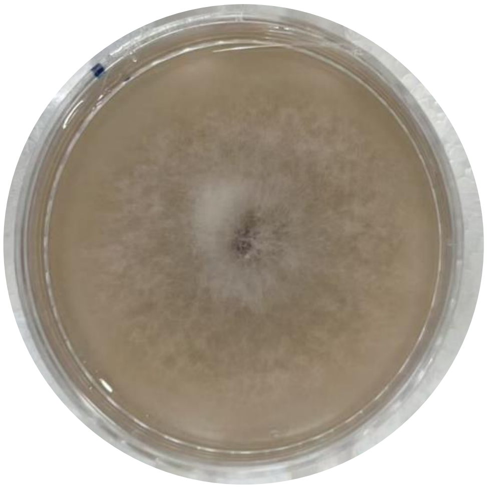

Figure S1. Colony morphology of *Aspergillus ochraceopetaliformis* FM451 cultured on PDA medium

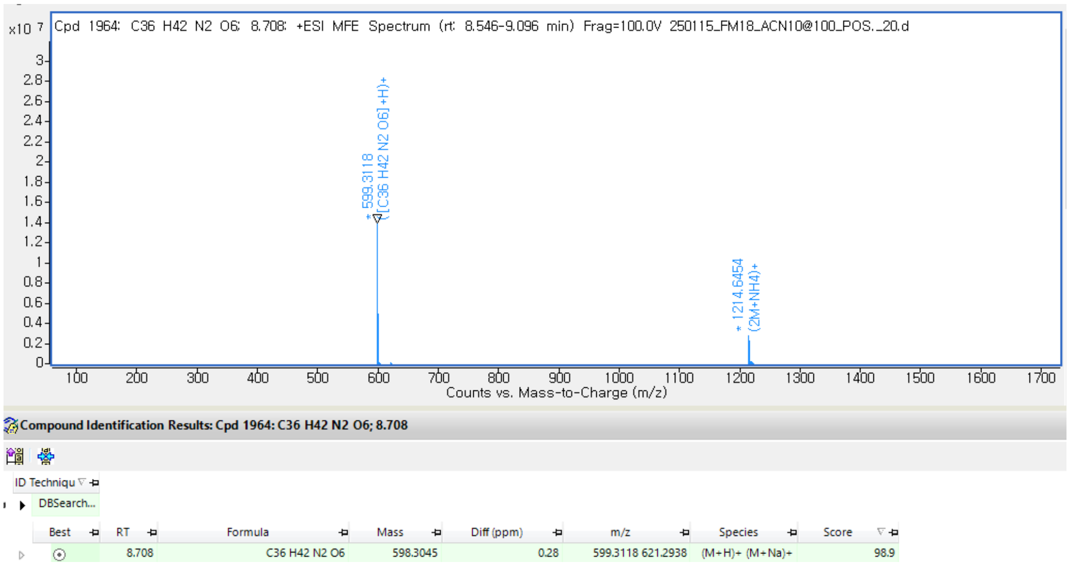

Figure S2. HR-ESIMS data of compound **1**

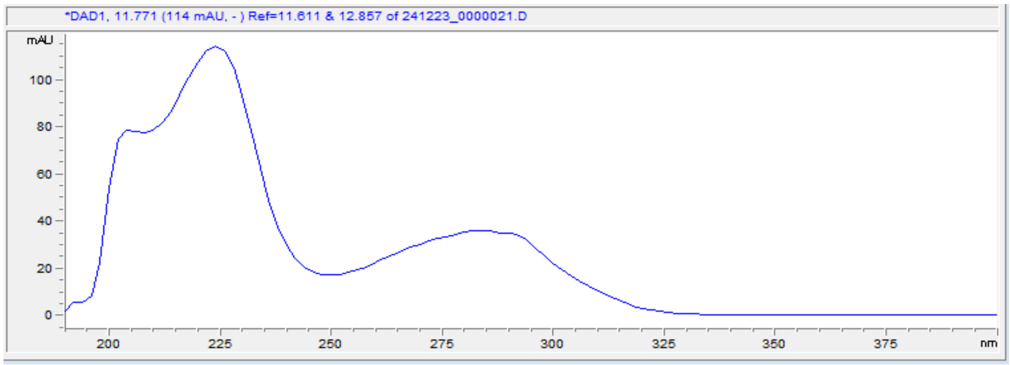

Figure S3. UV chromatogram of LC/MS for compound **1**

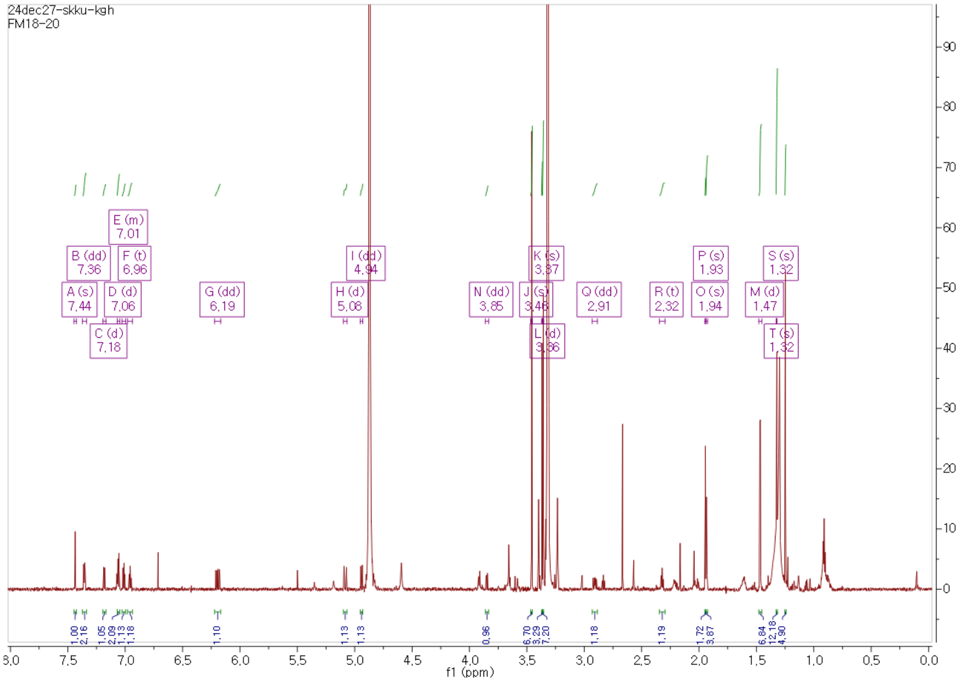

Figure S4. ^{1}H-NMR (CD_3OD, 850 MHz) spectrum of compound **1**

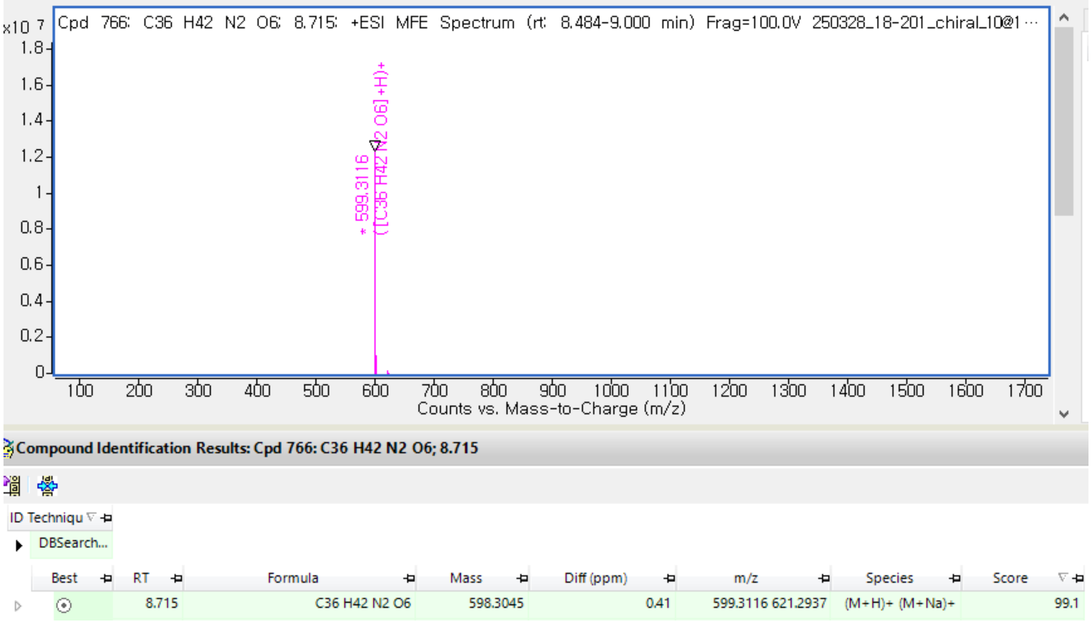

Figure S5. HR-ESIMS data of compound **1a**

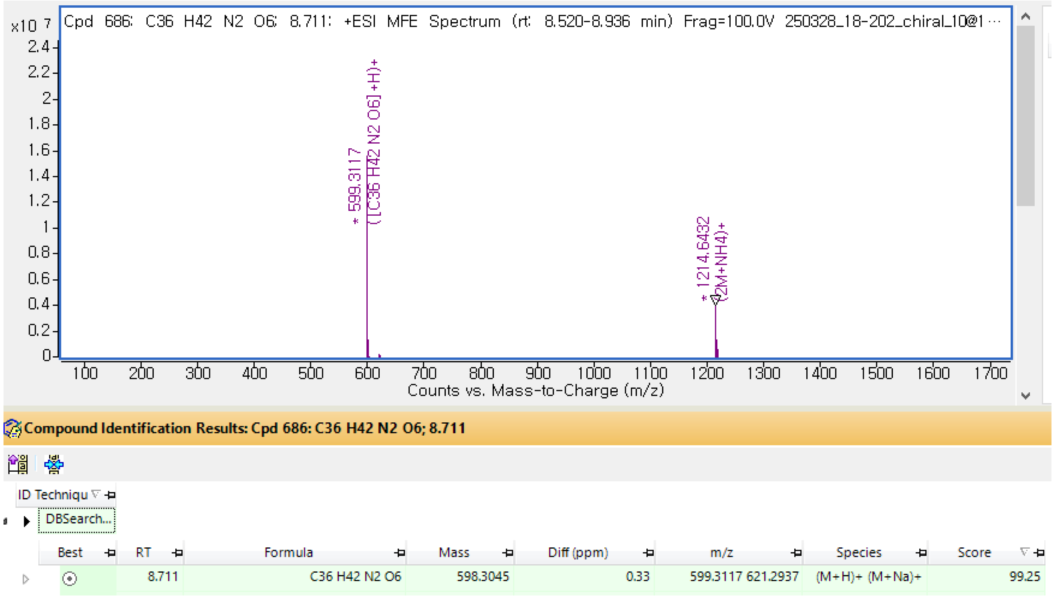

Figure S6. HR-ESIMS data of compound **1b**

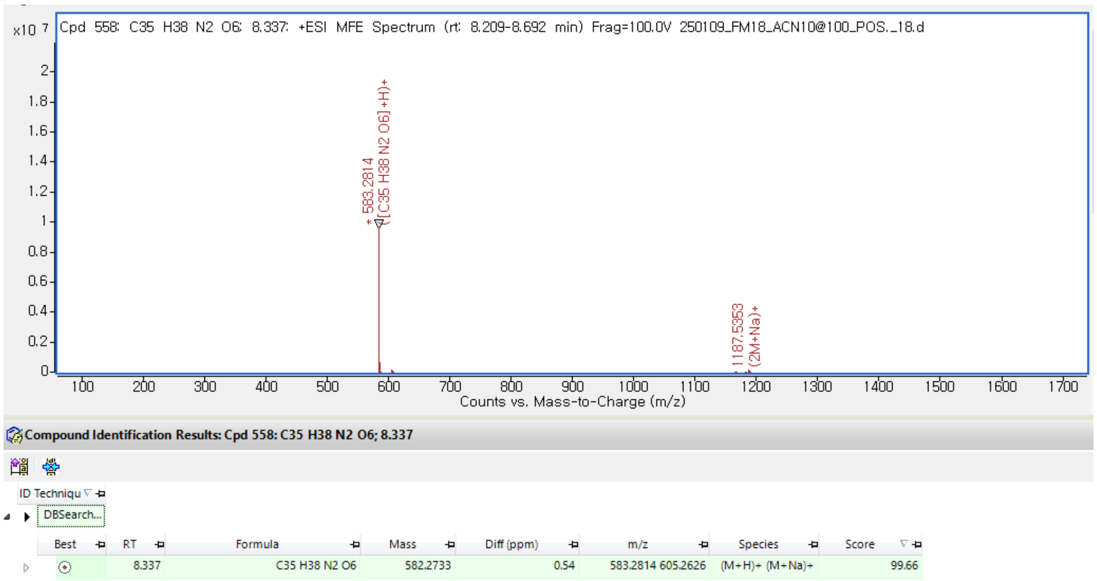

Figure S7. HR-ESIMS data of compound **2**

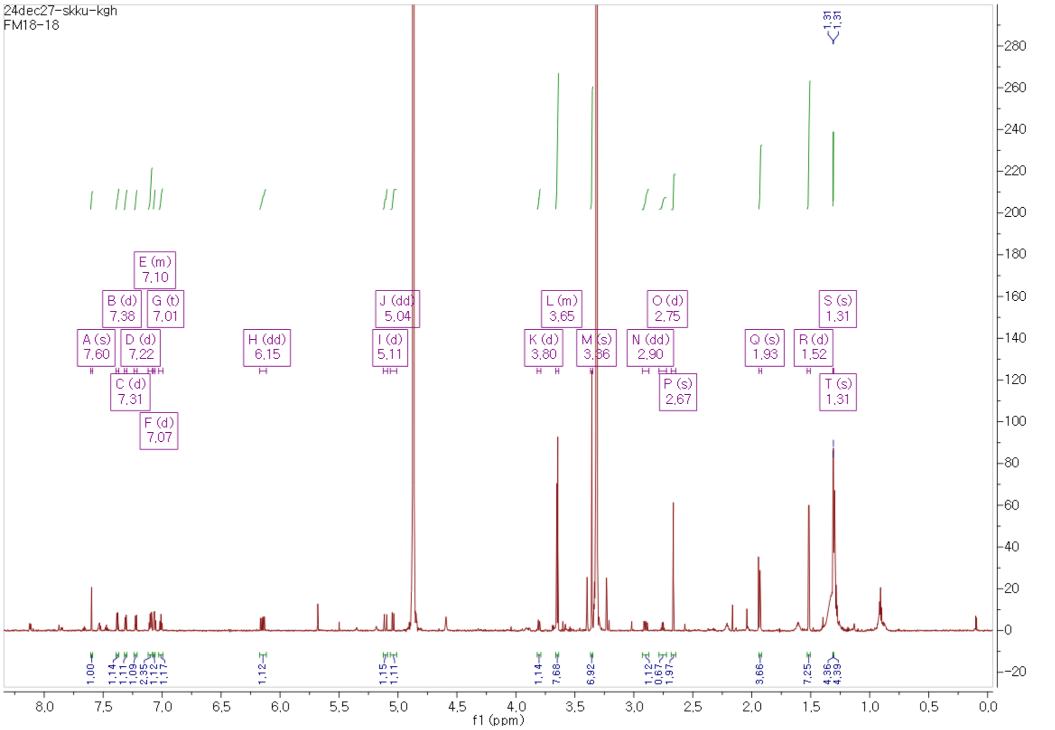

Figure S8. ^{1}H NMR spectrum (CD_3OD, 850 MHz) of compound **2**

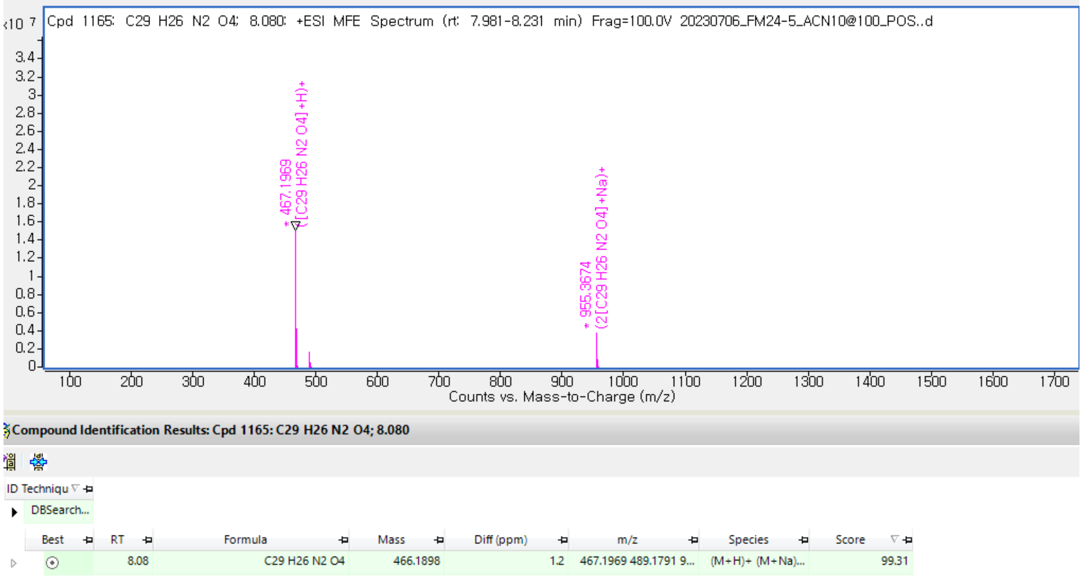

Figure S9. HR-ESIMS data of compound **3**

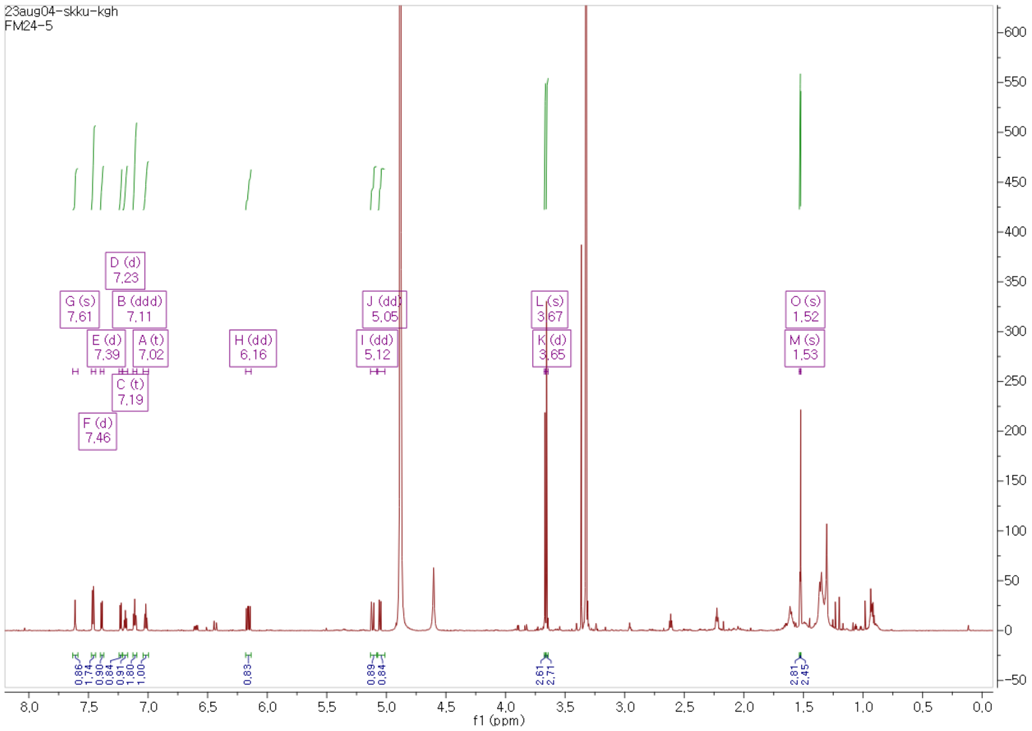

Figure S10. ^{1}H NMR spectrum (CD_3OD, 850 MHz) of compound **3**

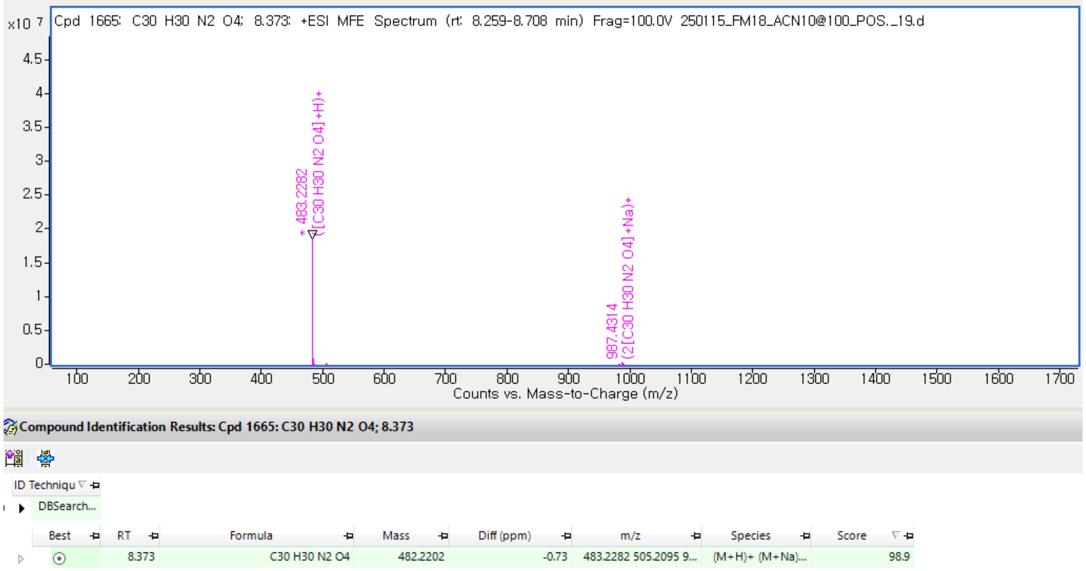

Figure S11. HR-ESIMS data of compound **4**

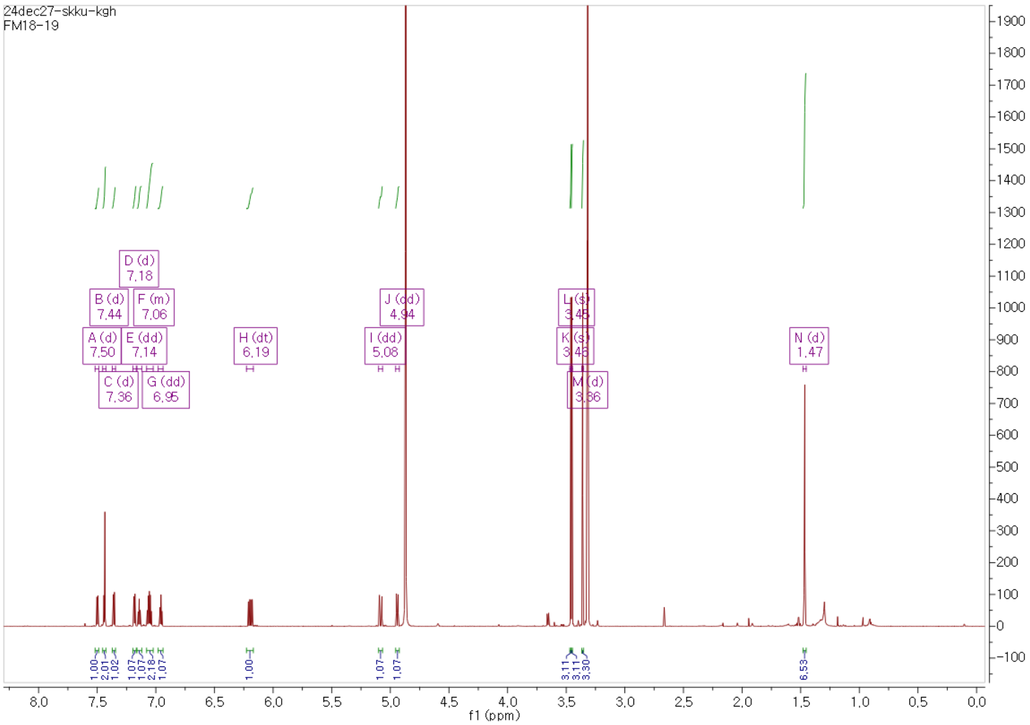

Figure S12. ^{1}H NMR spectrum (CD_3OD, 850 MHz) of compound **4**

(**A**) Total ion chromatogram (TIC) of the crude extract

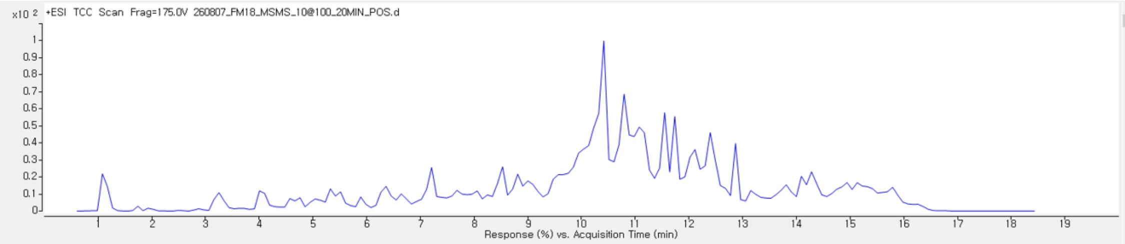

(**B**) Extracted ion chromatogram (EIC) at *m/z* 583.2814 for compound **2**

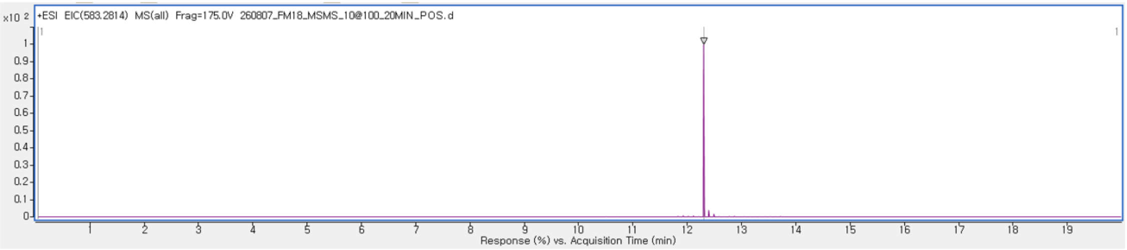

(**C**) HRMS spectrum of peak at 12.301 min in the crude extract

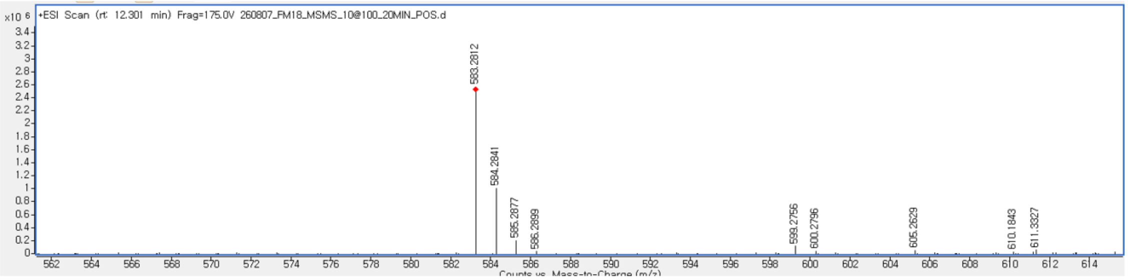

(**D**) HRMS spectrum of compound **2**

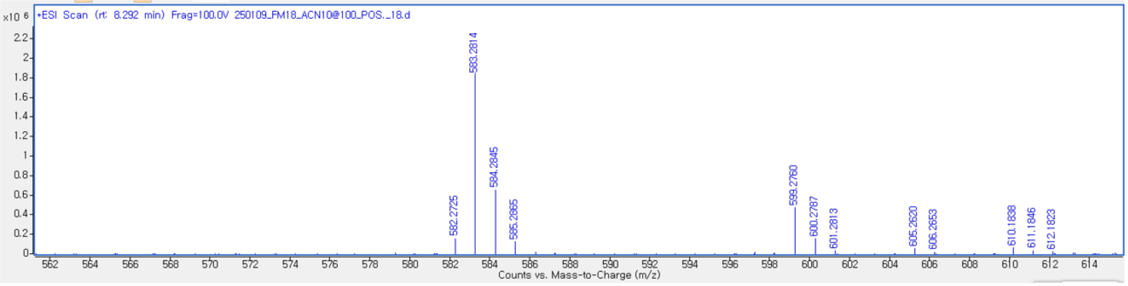

Figure S13. LC–HRMS analysis of compound **2** in the crude extract of *A. ochraceopetaliformis* FM451. (A) Total ion chromatogram (TIC) of the crude extract. (B) Extracted ion chromatogram (EIC) at *m/z* 583.2814 for compound **2**. (C) HRMS spectrum of peak at 12.301 min in the crude extract, and (D) HRMS spectrum of compound **2**.
